# Application of antibiotic bone cement in the treatment of infected diabetic foot ulcers in type 2 diabetes

**DOI:** 10.1186/s12891-023-06244-w

**Published:** 2023-02-21

**Authors:** Jiezhi Dai, Yu Zhou, Shasha Mei, Hua Chen

**Affiliations:** 1 Department of Orthopedic Surgery, Shanghai Jiao Tong University Affiliated Sixth People’s Hospital, Shanghai, China; 2 Department of Orthopedic Surgery, Civil Aviation Hospital of Shanghai, Shanghai, China; 3 Department of Anesthesiology, Shanghai Jiao Tong University Affiliated Sixth People’s Hospital, Shanghai, China

**Keywords:** DFU, Infection, Debridement, Antibiotic bone cement, Limb salvage

## Abstract

**Background:**

In this study, we try to investigate the effect of antibiotic bone cement in patients with infected diabetic foot ulcer (DFU).

**Methods:**

This is a retrospective study, including fifty-two patients with infected DFU who had undergone treated between June 2019 and May 2021. Patients were divided into Polymethylmethacrylate (PMMA) group and control group. 22 patients in PMMA group received antibiotic bone cement and regular wound debridement, and 30 patients in control group received regular wound debridement. Clinical outcomes include the rate of wound healing, duration of healing, duration of wound preparation, rate of amputation, and frequency of debridement procedures.

**Results:**

In PMMA group, twenty-two patients (100%) had complete wound healing. In control group, twenty-eight patients (93.3%) had wound healing. Compared with control group, PMMA group had fewer frequencies of debridement procedures and shorter duration of wound healing (35.32 ± 3.77 days vs 44.37 ± 7.44 days, P < 0.001). PMMA group had five minor amputation, while control group had eight minor amputation and two major amputation. Regarding the rate of limb salvage, there was no limb lose in PMMA group and two limb losses in control group.

**Conclusion:**

The application of antibiotic bone cement is an effective solution for infected DFU treatment. It can effectively decreased the frequency of debridement procedures and shorten the healing duration in patients with infected DFU.

## Key messages

In this study, we try to investigate the effect of antibiotic bone cement in patients with infected diabetic foot ulcer. Patients treated with antibiotic bone cement were included in this retrospective study. Antibiotic bone cement treatment can effectively decreased the frequency of debridement procedures and shorten the healing duration in patients with infected DFU.

Diabetic foot ulcer (DFU) is a common complication of diabetes mellitus [1]. Impaired wound healing in diabetic patients can lead to infections, chronic ulcers with a recurrence rate of 66% and even lower extremity amputation, which significantly affects the patients’ quality of life [2].

Current treatment guidelines for DFU recommend foot wound debridement, glycemic control, infection management, revascularization, and decompression to promote healing [3]. Wound infection is a predictor of poor wound healing and amputation [4]. It can develop and spread rapidly and cause significant and irreversible tissue damage. A correct understanding of diabetic foot infection and the application of antibiotic treatment are the key to improve the efficacy. With the main advantage of high drug concentration on the target site and low risk of systemic toxicity, local antibiotic therapy acts as an effective treatment [5]. Antibiotic bone cement has been widely used in the treatment of infected arthroplasty and osteomyelitis [6, 7]. It has the dual function of preventing soft tissue contracture and delivering antibiotics locally to bone and soft tissue by elution [8]. Polymethylmethacrylate (PMMA) is the major bone cement material in the orthopedic procedures. There are few similar studies on the application of antibiotic bone cement as an effective method for infected diabetic foot ulcer. In this present study, we retrospectively reviewed our experience on the use of antibiotic bone cement for infected DFU treatment.

## Materials and methods

### Study design

This is a single-center, retrospective study including patients with infected DFU treated from June 2019 to May 2021. Patients who meet the following inclusion and exclusion criteria were recruited. This retrospective study was approved by our institutional review board.

### Inclusion criteria

Type 2 diabetes.

An ankle-brachial index (ABI) > 0.7, and at least one of the anterior tibial artery, posterior tibial artery, and peroneal artery can reach the level of the ankle joint.

Infected diabetic foot was defined as Grade IIIB or Grade IVB according to the Texas University Classification [9] and Grade 2, 3 or 4 according to the Wagner classification [10]. Diagnosis is based on patient’s history, clinical sign, radiographic examination, laboratory evaluation and positive bacterial culture. We apply preoperative X-ray, bone biopsies and surgeon’s experience to judge whether there is osteomyelitis.

### Exclusion Criteria

Patients with chronic wound due to vasculitis, pyoderma gangrenosum, pressure ulcer, or wound infections not related to DM; known or suspect malignancy of current ulcer; currently undergoing radiation or chemotherapy [11].

Patients were split into two groups based on the surgical procedure. Patients treated with regular wound debridement were defined as control group. The PMMA group included patients who received antibiotic bone cement and regular wound debridement.

### Medical care

Preoperative and postoperative medical care was the same for both groups of patients, except for the different surgical procedures. Appropriate medical treatment included blood glucose regulation, perfusion improvement by prostaglandins or antiplatelet drugs, appropriate antibiotics administration, and routine sterile dressing change. Tissue samples were taken for microbiological analysis. Sensitive antibiotics were selected for intravenous application according to the results of drug susceptibility test. It was switched to oral therapy when the patients was clinically improving. During the treatment period, blood glucose was monitored daily, and oral hypoglycemic drugs, such as metformin, acarbose, etc., were used. To control blood glucose, subcutaneous injection of short-acting and long-acting insulin were applied, and the dose was dynamically adjusted until the wound healed.

### Surgical procedures

For PMMA group, treatment was divided into two stages: the first to treat the diabetic wound infection and the second to reconstruct the wound defect. In debridement, we removed and debrided nonviable infected soft tissues and bones. The edges of debridement were achieved until the soft tissues and bones presented generally healthy. After thorough soft tissue and bone debridement, the wound was covered with antibiotic cement. We used PMMA (Smith & Nephew, TN, USA) premixed with gentamicin and added vancomycin to the powder (2 g vancomycin per 40 g mix) before mixing the powder and liquid. The wound was covered with gauze dressings and changed every two days.

Two weeks after PMMA implantation, we removed the antibiotic cement. The second stage surgery that reconstructing the soft tissue defect was conducted when there were no clinical signs and symptoms of infection. Otherwise, further debridement and PMMA implantation were performed. It depended on their clinical signs, laboratory evaluation and clinical experience of surgeons. The standard to decide when to perform the reconstructive procedures included that the wound was fresh enough, the bacterial culture was negative, the blood glucose was well controlled, and there was no or mild anemia, etc. Soft tissue defect was reconstructed with skin grafting, skin flap coverage or closed primarily. After wound healing, we continued to follow the patients monthly for three consecutive visits.

For control group, after primary debridement, the wound was covered with the negative pressure wound therapy system (VSD Medical Science and Technology Co. Ltd., Wuhan, China). This device promoted wound healing by removing fluid from open wounds, preparing the wound bed for closure, reducing edema, and promoting formation and perfusion of granulation tissue [12]. Patients received continuous debridement weekly until clinical signs and infectious symptoms were free. Soft tissue defect was reconstructed with skin grafting, skin flap coverage or closed primarily.

### Outcomes

Clinical outcomes include the rate of wound healing, healing duration, rate of amputation, and frequency of debridement procedures. We defined the healing rate as the percentage of patients whose wounds healed at a given time point (wound size of 0 cm and Wagner score of 0 for each wound). The amputation rate is defined as the percentage of patients who lost a limb or a part of it at a given time point [13]. The indication of amputation included (1) all efforts to treat progressive diabetic foot infection remain insufficient, (2) progressive necrosis or gangrene, (3) intractable pain, and (4) acute arterial occlusion. Minor amputation is defined as below level of the ankle, which require the preservation of a functional foot to stand and walk without a prosthesis [14]. Major amputation refers to above, through or below knee loss of a limb, and represents failed limb salvage [15].The duration of healing is defined as the time from the initial surgery to complete wound healing. The duration of wound preparation is defined as the time from initial surgery to reconstructive procedures. Clinical evaluation of infection symptoms include swelling, exudate, odor, surrounding cellulitis, tissue necrosis, etc. [16].

Statistical analysis is performed with SPSS 18.0 software in this study. Data are expressed as means and standard deviations. Differences between groups are assessed with Student’s t-test or Mann–Whitney U test. The frequencies of the data are evaluated with Fisher’s exact test. The value is assumed to be significant at p-value < 0.05.

## Results

**Fig. 1:**
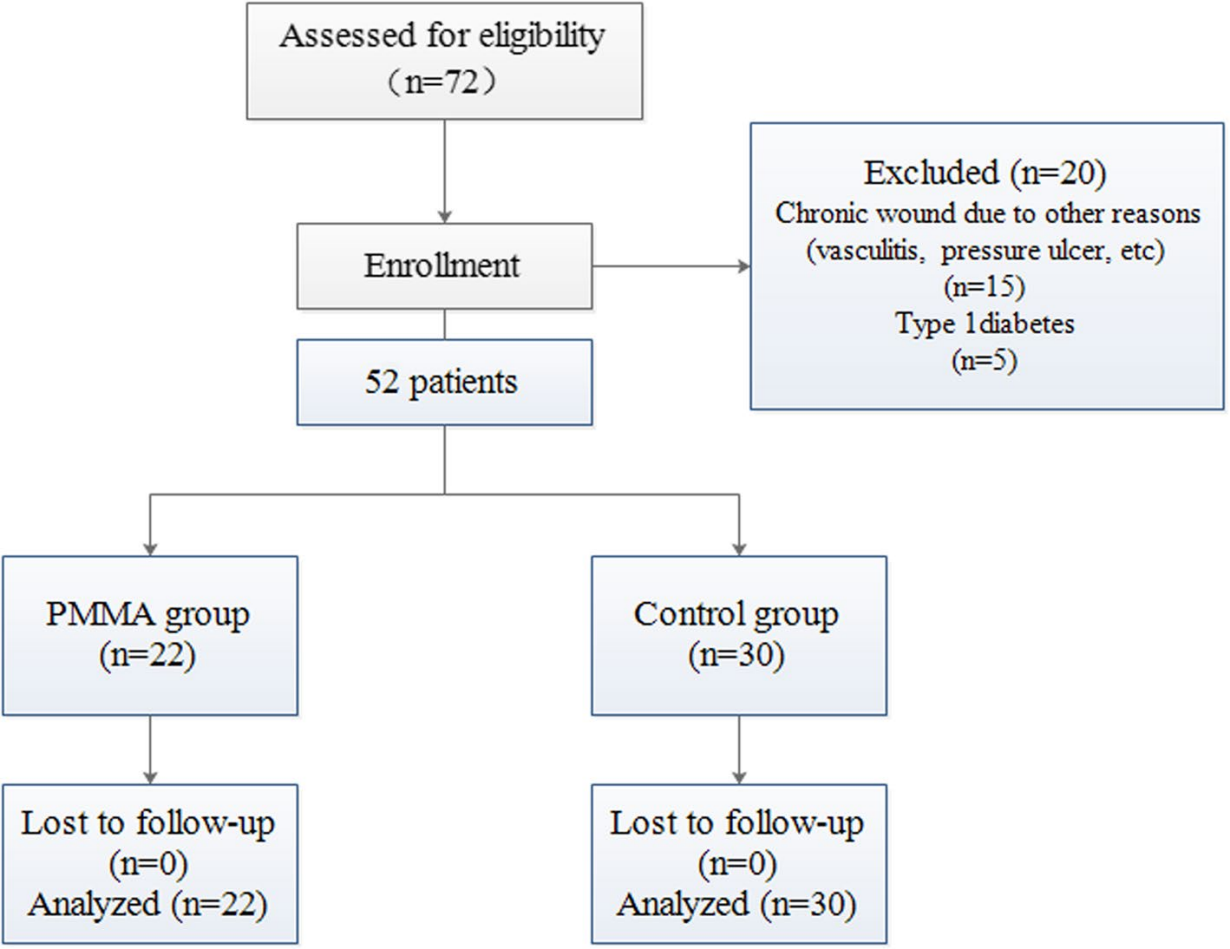
The flow diagram of the patient selection process

After the assessment of 72 patients, 20 patients did not meet the study selection criteria and were excluded and 52 patients were included to the study (Fig. 1). The demographics data of the included study population were presented in Table 1. In this study, twenty-two patients with infected DFU (9 males and 13 females, aged 52.81 ± 9.78 years) were enrolled in PMMA group. Thirty patients with infected DFU (11 males and 19 females, aged 54.83 ± 8.64 years) were enrolled in control group. We also assessed the severity of any diabetic foot infection using the Infectious Diseases Society of America/International Working Group on the Diabetic Foot classification scheme and all included patients were moderate infection (grade 3 or grade 3(O)). There was no significant difference between the two groups in age, gender, BMI, Fasting blood glucose, HbA1c, SCr, BUN, and ABI (*P* > 0.05). Patients in both groups were type 2 diabetes mellitus. Table 2 presented the clinical data for each patient.

**Table 1:** Clinical characteristics of the study

	PMMA	Control	*P* value
Number	22	30	
Age (years)	52.81 ± 9.78	54.83 ± 8.64	0.436
Gender (Male/Female)	9/13	11/19	
DM duration (years)	10.95 ± 4.51	12.77 ± 4.94	0.182
Fasting blood glucose (mmol/L)	9.06 ± 1.80	9.68 ± 1.63	0.202
HbAlc (%)	10.11 ± 1.53	9.54 ± 1.34	0.158
BMI (kg/m2)	23.94 ± 1.35	24.70 ± 1.52	0.069
SCr (μmol/L)	71.68 ± 25.95	79.63 ± 24.69	0.267
BUN (μmol/L)	4.62 ± 1.27	4.99 ± 1.60	0.370
ABI	0.93 ± 0.13	0.91 ± 0.12	0.629
Wagner Classification			
Grade 2	8	12	
Grade 3	10	12	
Grade 4	4	6	
The international classification of the infection of DFU			
Grade 3	16	23	
Grade 3(O)	6	7	

*SCr* Serum creatinine, *BUN* Blood urea nitrogen, *ABI* Ankle brachial index

**Table 2:** Summary patient data

Case	Gender	Age	surgical procedure	Ulcer location	Wound duration (days)	Complicated with osteomyelitis (Yes/No)	Reconstruction method	Duration of healing (days)	Duration of wound preparation (days)
1	Female	45	PMMA	Dorsal midfoot	24	No	Skin graft	32	16
2	Female	52	PMMA	Dorsal forefoot	41	No	Skin graft	35	19
3	Male	48	PMMA	4th,5th toes	16	No	Direct closure	29	10
4	Female	43	PMMA	Plantar hindfoot	35	No	Flap coverage	42	28
5	Female	45	PMMA	Dorsal midfoot	32	No	Skin graft	35	18
6	Female	72	PMMA	2nd toe	55	Yes	Direct closure	33	18
7	Female	46	PMMA	Plantar midfoot	29	No	Skin graft	36	24
8	Male	52	PMMA	Hallux toe	31	Yes	Direct closure	36	25
9	Male	66	PMMA	3rd,4th,5th toes and dorsal	47	Yes	Flap coverage	40	21
10	Female	50	PMMA	3rd toe	22	No	Direct closure	30	14
11	Female	41	PMMA	Dorsal midfoot	23	No	Skin graft	36	24
12	Male	64	PMMA	Lateral ankle	45	No	Flap coverage	39	20
13	Male	63	PMMA	Plantar hindfoot	36	No	Direct closure	37	28
14	Female	40	PMMA	Dorsal midfoot	37	No	Skin graft	36	24
15	Male	70	PMMA	Plantar forefoot	19	No	Direct closure	28	10
16	Male	59	PMMA	Plantar hindfoot	44	No	Flap coverage	39	19
17	Female	49	PMMA	2nd,3rd,4th toes	34	Yes	Direct closure	35	19
18	Female	65	PMMA	Hallux toe	18	No	Direct closure	32	15
19	Male	51	PMMA	Hallux and 2nd toes	29	Yes	Direct closure	36	20
20	Female	52	PMMA	Lateral dorsal forefoot	40	No	Direct closure	36	19
21	Male	44	PMMA	Dorsal midfoot	34	No	Skin graft	42	29
22	Female	45	PMMA	5th toe and lateral dorsal	51	Yes	Direct closure	33	16
23	Male	64	Control	Dorsal midfoot	17	No	Skin graft	47	30
24	Female	40	Control	2nd,3rd toes	22	No	Direct closure	47	33
25	Male	51	Control	Lateral dorsal foot	21	No	Direct closure	28	10
26	Female	67	Control	Dorsal forefoot	46	No	Skin graft	53	37
27	Female	48	Control	2nd,4th toes	25	Yes	Direct closure	40	23
28	Male	40	Control	Lateral Forefoot	19	No	Direct closure	45	33
29	Female	55	Control	Medial forefoot	27	No	Direct closure	47	34
30	Female	60	Control	Dorsal foot	33	No	Skin graft	51	34
31	Male	54	Control	Hallux toe	24	No	Direct closure	48	34
32	Male	45	Control	hindfoot	53	No	Flap coverage	56	38
33	Female	52	Control	Dorsal forefoot	28	No	Skin graft	45	28
34	Male	53	Control	Dorsal midfoot	55	Yes	Flap coverage	50	30
35	Male	56	Control	Hindfoot	24	No	Flap coverage	49	31
36	Female	68	Control	hindfoot	26	Yes	Flap coverage	56	36
37	Female	59	Control	3rd,4th,5th toes	34	Yes	Direct closure	54	43
38	Female	65	Control	Dorsal forefoot	21	No	Skin graft	37	20
39	Male	44	Control	Hallux, 2nd,3rd toes	24	Yes	Direct closure	41	29
40	Female	40	Control	Dorsal forefoot	17	No	Skin graft	32	15
41	Female	62	Control	Dorsal midfoot	27	No	Flap coverage	50	29
42	Female	61	Control	Hallux toe	24	No	Direct closure	33	22
43	Male	51	Control	Plantar midfoot	37	No	Direct closure	49	34
44	Male	54	Control	Dorsal forefoot	25	No	Skin graft	43	27
45	Female	54	Control	Dorsal midfoot	19	No	Skin graft	43	25
46	Female	62	Control	4th,5th toes	17	No	Direct closure	30	14
47	Female	49	Control	Dorsal forefoot	25	No	Skin graft	36	18
48	Female	63	Control	5th toe	19	No	Direct closure	42	30
49	Male	50	Control	4th,5th toes and dorsal forefoot	23	Yes	Flap coverage	51	32
50	Female	45	Control	Medial midfoot	16	No	Skin graft	41	25
51	Female	69	Control	Forefoot and midfoot	43	Yes	Major amputation	42	25
52	Female	64	Control	Hallux toe and ankle	35	Yes	Major amputation	45	27

**Fig. 2:**
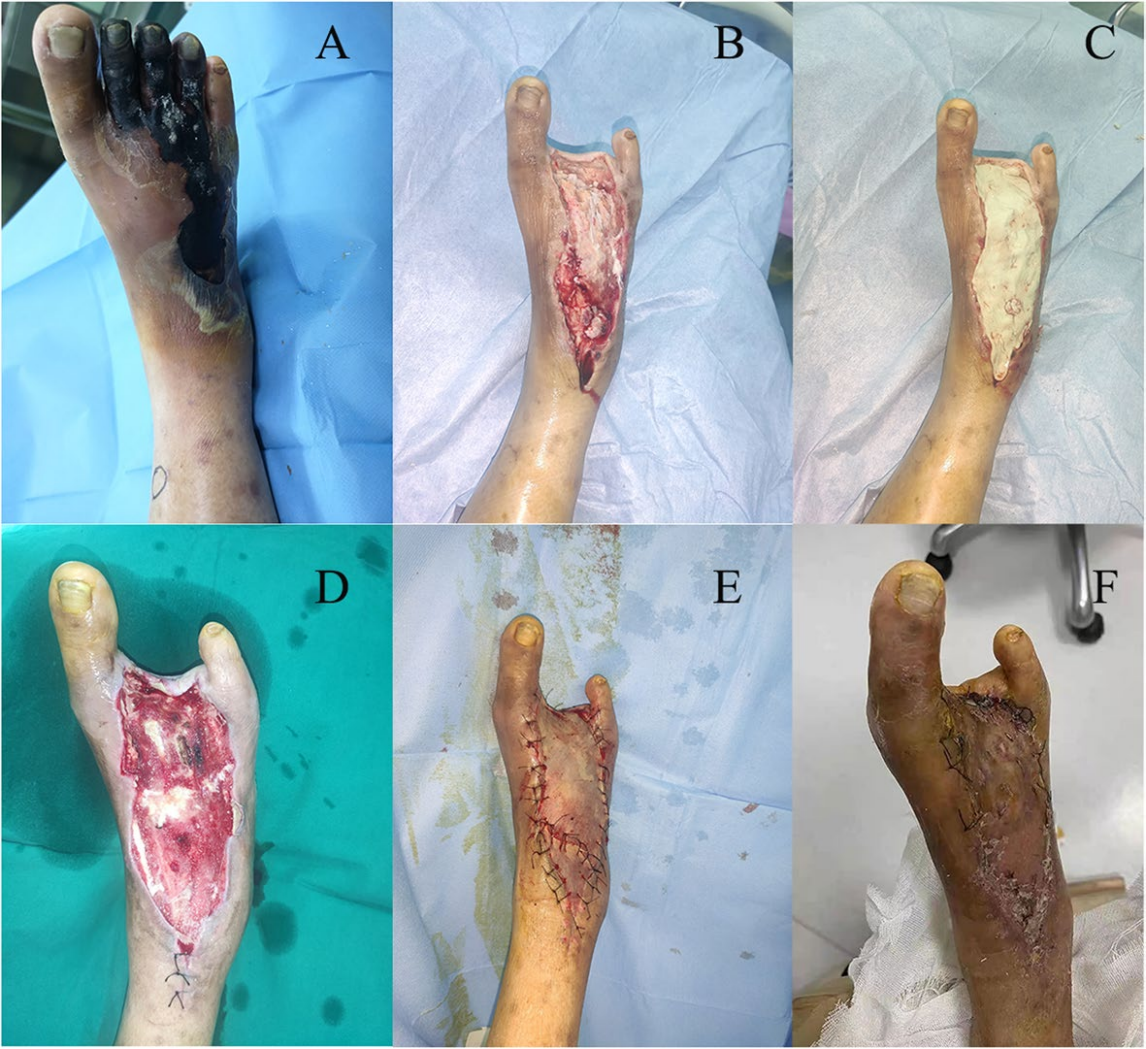
Clinical case: female, 52 years old, Wagner grade 4. **A** Initial wound before surgical debridement; **B** The nonviable, infected soft tissues and necrotic toes were debrided; **C** The defect was filled with antibiotic bone cement; **D** Antibiotic bone cement was removed after 2 weeks; **E** The wound was covered with skin grafting; **D** The wound was completely healed at follow-up

**Fig. 3:**
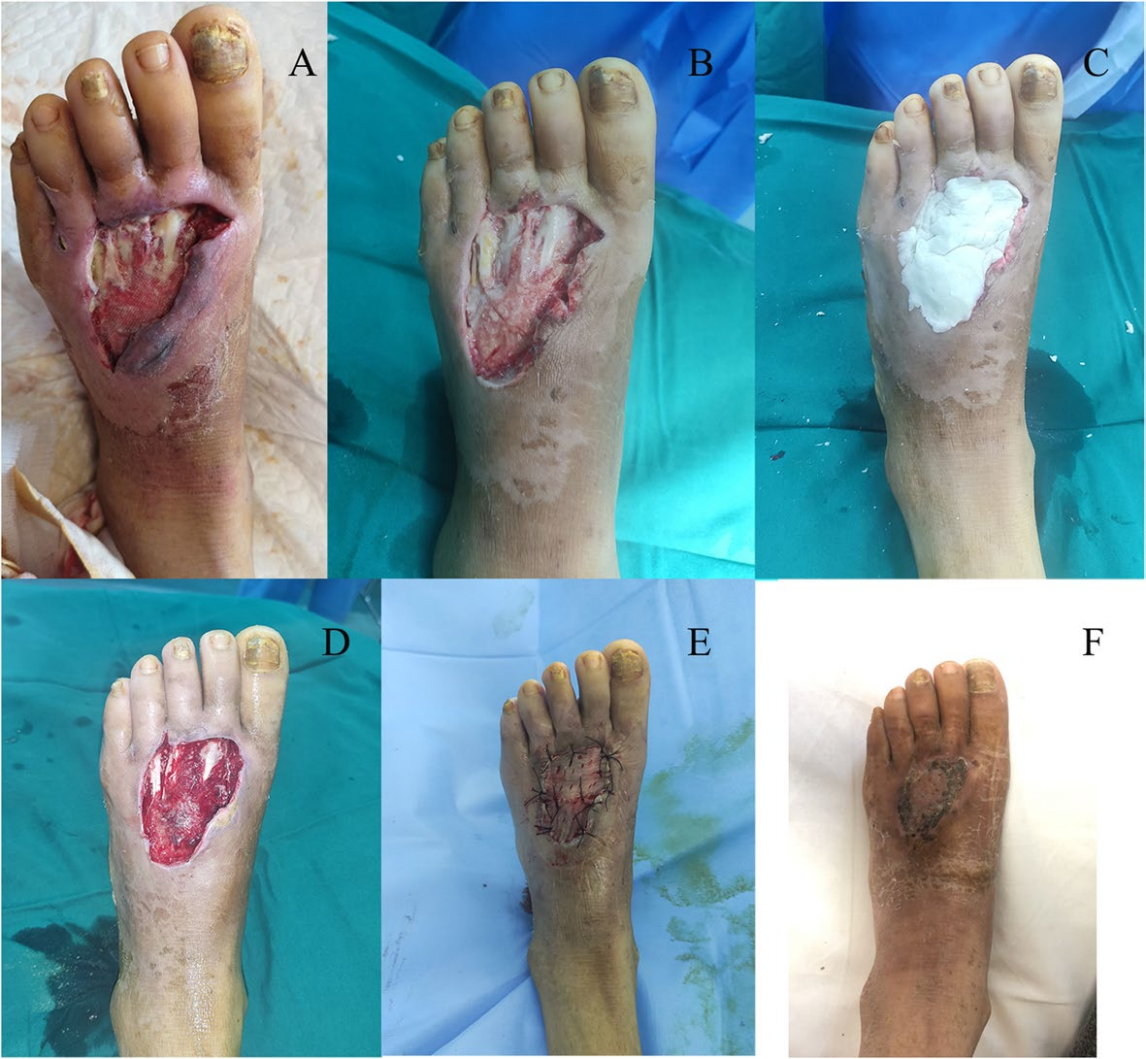
Clinical case: female, 45 years old, Wagner grade 2. **A** Initial wound before surgical debridement; **B** The nonviable and infected soft tissues were debrided; **C** The defect was filled with antibiotic bone cement; **D** Antibiotic bone cement was removed after 2 weeks; **E** The wound was covered with skin grafting; **D** The wound was completely healed at follow-up

The clinical outcomes were presented in Table 3. In PMMA group, twenty-two patients (100%) had complete wound healing (Figs. 2 and 3). The mean of wound healing time was 35.32 ± 3.77 days with the average number of debridement procedures of 1.50 ± 0.51. The mean time of wound preparation in PMMA groups was 19.82 ± 5.29 days. Minor amputation was reported in five patients (22.7%). In control group, twenty-eight patients (93.3%) had wound healing. The mean duration of wound healing was 44.37 ± 7.44 days with the average number of debridement procedures of 2.13 ± 0.86. The mean time of wound preparation in control groups was 28.20 ± 7.53 days. There were eight minor amputation (26.7%) and two major amputation (6.7%) in control group. With regard to the rate of limb salvage, there was no limb lose in PMMA group and two limb loss in control group. None of patients reported ulcer recurrence in 3 months’ follow-up.

**Table 3:** Clinical outcomes between PMMA group and control group

	PMMA	Control	*P* value
Number of healing	22	28	0.502
Minor amputation	5	8	1.000
Major amputation	0	2	0.502
Duration of healing (days)	35.32 ± 3.77	44.37 ± 7.44	< 0.001
Duration of wound preparation (days)	19.82 ± 5.29	28.20 ± 7.53	< 0.001
Frequency of debridement procedures	1.50 ± 0.51	2.13 ± 0.86	0.003

Wound microflora pathogens isolated were presented in Table 4. *Staphylococcus aureus* is the most prevalent genera that isolated pathogens in both groups, followed by *Escherichia coli*, *Enterococcus faecalis*, and *Enterobacter cloacae*. There was no significant difference in cultivation results between both groups.

**Table 4:** Wound microflora pathogens isolated

	PMMA	Control
*Staphylococcus aureus*	10	14
*Escherichia coli*	7	8
*Enterococcus faecalis*	3	4
*Enterobacter cloacae*	2	4

## Discussion

In this retrospective study, we found that antibiotic bone cement treatment can effectively decreased frequency of debridement procedures and shorten the healing duration in patients with infected DFU. It is effective as an adjunct to extensive debridement for salvage of infected DFU and reduces the probability of amputation in patients.

Debridement involves removing all devitalised, contaminated or foreign material in or near the wound until surrounding healthy tissue is shown and it is widely used in diabetic foot care [17]. It plays a key role in infection control, and speeds the healing process in most patients with diabetic foot wounds. If progressive tissue necrosis or further deep infection occurs, surgical debridement should be repeated. Piaggesi et al. evaluated the efficacy of DFU surgical debridement compared to conventional non-surgical management. Compared with conventional treatment, surgical debridement has proved to be an effective methods for DFU patients in terms of healing time, complications, and recurrence [18].

Even with the well-established principles to managing DFU, there is still room for improvement in DFU treatment. Since the first report in 1970, PMMA-based antibiotic bone cement system has been extensively studied in the treatment of osteomyelitis and in the prevention of artificial hip/knee replacement-associated bone infection [19]. As we all known, the release of antibiotic in the antibiotic bone cement to control local infection is achieved by direct dissolution at the surface and diffusion from the bulk. In a large prospective study of DFU patients, the presence of infection was associated with a 50% increased risk of minor amputation compared to ulcer patients without infection [20]. The application of antibiotic bone cement to deliver antibiotic in patients with diabetic foot infection might be an effective adjuvant therapy.

In PMMA group, the mean of wound healing time was 35.10 ± 3.61 with the average number of debridement procedures of 1.55 ± 0.51 while the mean duration of wound healing was 44.37 ± 7.44 with the average number of debridement procedures of 2.06 ± 0.88 in control group. The mean time of wound preparation in PMMA groups was shorter when compared with it in control groups (19.82 ± 5.29 days vs 28.20 ± 7.53 days). The antibiotic bone cement treatment can effectively decreased frequency of debridement procedure and shorten healing duration in diabetic patients with foot infection. Regard with the rate of limb salvage, there was no limb lose in PMMA group and two limb losses in control group. Limb salvage according to “Recommended standards for reports dealing with lower extremity ischemia” is applicable to the treatment results of interventions aimed at avoiding major amputation [21]. It might be an effective adjunct to extensive debridement for limb salvage.

There is no reliable evidence on the priority selection of effective antibiotic type according to the existing clinical practice guidelines. Based on bacterial culture results and drug sensitivity of wound secretion, moderate and severe diabetic foot infection are typically treated from 2 to 4 weeks of intravenous antibiotic therapy with 4 to 6 weeks of bone infection treatment [22]. Once the clinical symptoms and signs of infections resolved, antibiotics can often be discontinued [23]. Usually, emergency surgery is needed to control infection. However, there is a time interval between specimen culture and pathogen identification. Therefore, it’s hard to get a cultured antibacterial spectrum before surgery. Gram-negative bacteria were more abundant in diabetic foot infection in warm-countries and the *Staphylococcus* is among the most prevalent genera that isolated pathogens in nearly every series in the literature [24, 25], as well as in our study. The most common mixed antibiotics are vancomycin and gentamicin. Vancomycin is a glycopeptide antibiotic that is primarily effective against gram-positive such as *Staphylococcus aureus*. Gentamicin is an aminoglycoside antibiotic and has broad-spectrum antimicrobial activity. The synergistic action of two antibiotics in bone cement has longer bactericidal activity than single antibiotic-loaded bone cement [26]. The coupling of a glycopeptide with an aminoglycoside covers both Gram-negative and Gram-positive bacteria [27].

Generally, it is worth noting that few studies have demonstrated the advantages of antibiotic bone cement in clinical treatment of infected DFU. Three similar studies such as Liu et al. [5], Ehya et al. [28] and Melamed et al. [6] have found the adjunctive antibiotic bone cement to improve the outcomes in surgically treated diabetic foot osteomyelitis or infected diabetic foot ulcer. Liu et al. reported that the healing duration was 13.1 ± 3.7 weeks in the PMMA group and 26.4 ± 7.8 weeks in the control group. The mean of healing time was 79.4 days (95% CI, 71–90) in the PMMA group and 101.7 days (95% CI, 93–110) in the control group in the study by Mendame. These findings are similar to our results that antibiotic bone cement can effectively shorten healing duration in patients with infected DFU. However, the average healing time in these studies are longer than ours. The prolonged wound preparation time may have contributed to the poor outcome. One of the key issues for the treatment of DFU is the prolonged wound healing time, which may have resulted from the prolonged wound preparation time. The wound preparation period is a very subjective process, and it is depended very much on the doctor’s will. In our study, it is still quite long from the beginning of the treatment to the healing. The standard to decide when to perform the reconstructive procedures included that the wound was fresh enough, the bacterial culture was negative, the blood glucose was well controlled, and there was no or mild anemia, etc. However, clinical signs such as granulation growth or freshness, wound exudation, etc. depend on the experience of the surgeon and may therefore influence the treatment options of the attending doctor. Standardization are needed to decide when to perform the reconstructive procedures.

In this study, we reviewed our experience on the management of diabetic foot infection by inclusion of patients with Texas classification IIIB and IVB or Wagner grades 2, 3 and 4 in the final analysis. The application of antibiotic bone cement on the defect from the surgical debridement of nonviable and infected soft tissue to treat infected DFU could achieve a satisfying medical outcome. The current outcomes should be assessed in light of some limitations, which mainly given that the analysis was retrospective. Larger and more prospective studies are still required to further evaluate these treatment option. In addition, all patients in the present study received vancomycin in the antibiotic bone cement. With continuous exploration and dialogue with infectious disease experts, it is more appropriate to decide which antibiotics to add according to the antibiotic sensitivity data.

During the application of antibiotic bone cement, some disadvantages should be concerned. It is worthy of note the surgeon waited for the antibiotic bone cement-mixed body temperature to drop significantly, in order to avoid exothermic heating of the surrounding tissues. In addition, the antibiotic bone cement may lead to poor wound drainage after it filled the residual dead space. We could make holes on the cement-mixed body during the last period of polymerization to promote drainage. Finally, the surgeon’s experience influences the final outcomes.

In conclusion, the application of antibiotic bone cement is an effective solution for infected DFU treatment. It can effectively decreased frequency of debridement and shorten healing duration in patients with infected DFU. However, more evidence studies are required to strengthen these conclusions.

## Data Availability

All data generated or analysed during this study are included in this published article.
